# Supplementary figures and images for: Klebsiella pneumoniae Reduces SUMOylation To Limit Host Defense Responses
Source: mBio. 2020 Sep 29;11(5):e01733-20. doi: 10.1128/mBio.01733-20 (PMC7527722; doi:10.1128/mBio.01733-20)

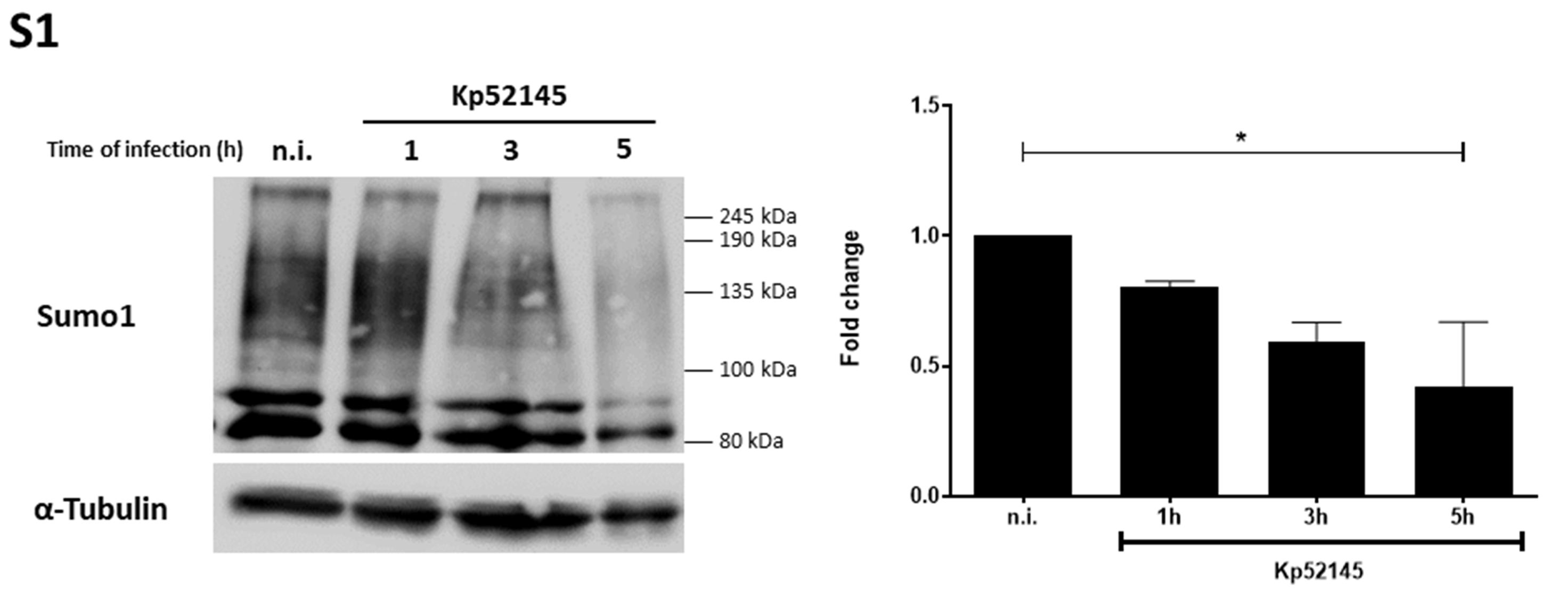

Supplement: FIG S1 [file mBio.01733-20-sf001.tif]

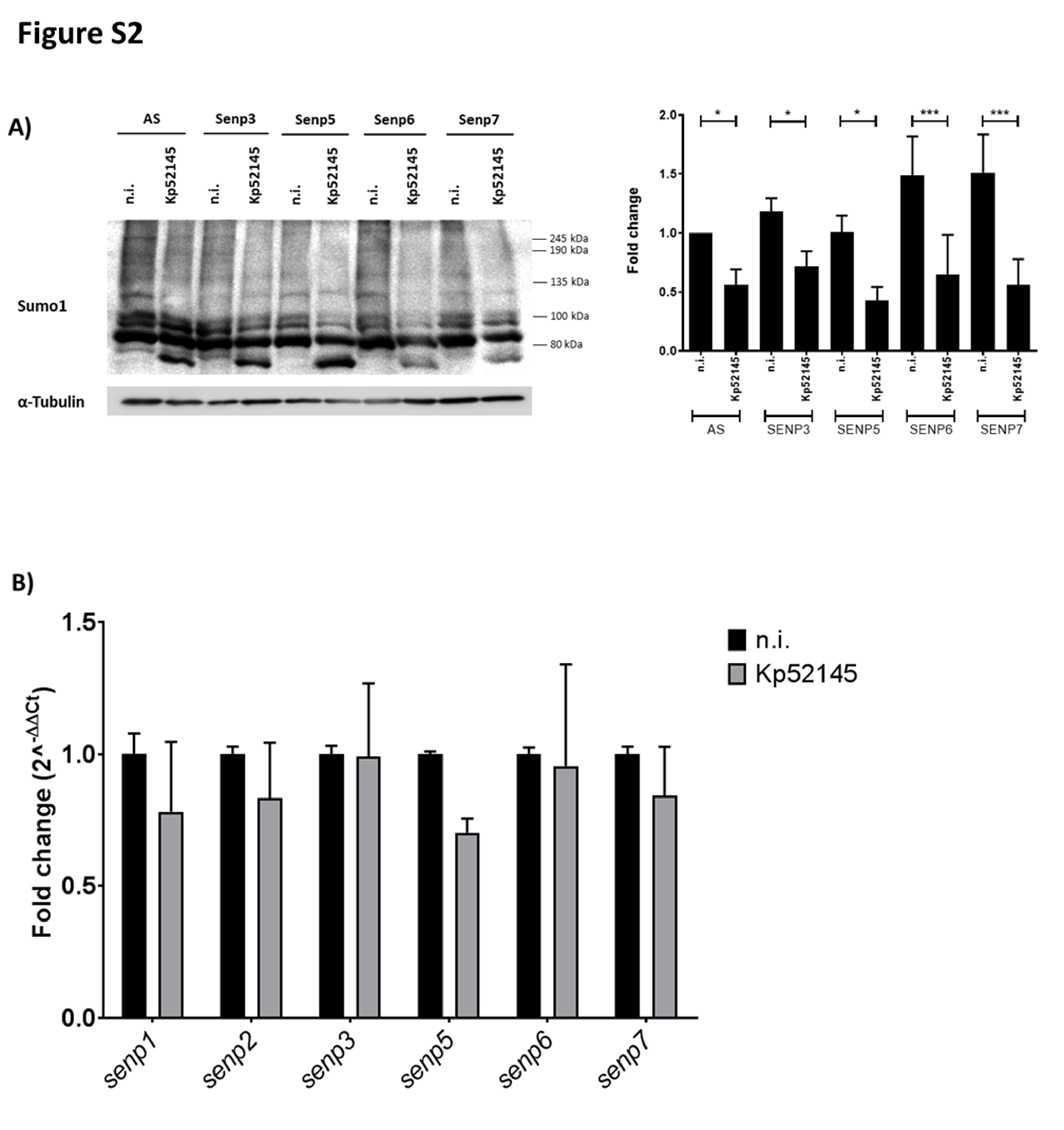

Supplement: FIG S2 [file mBio.01733-20-sf002.tif]

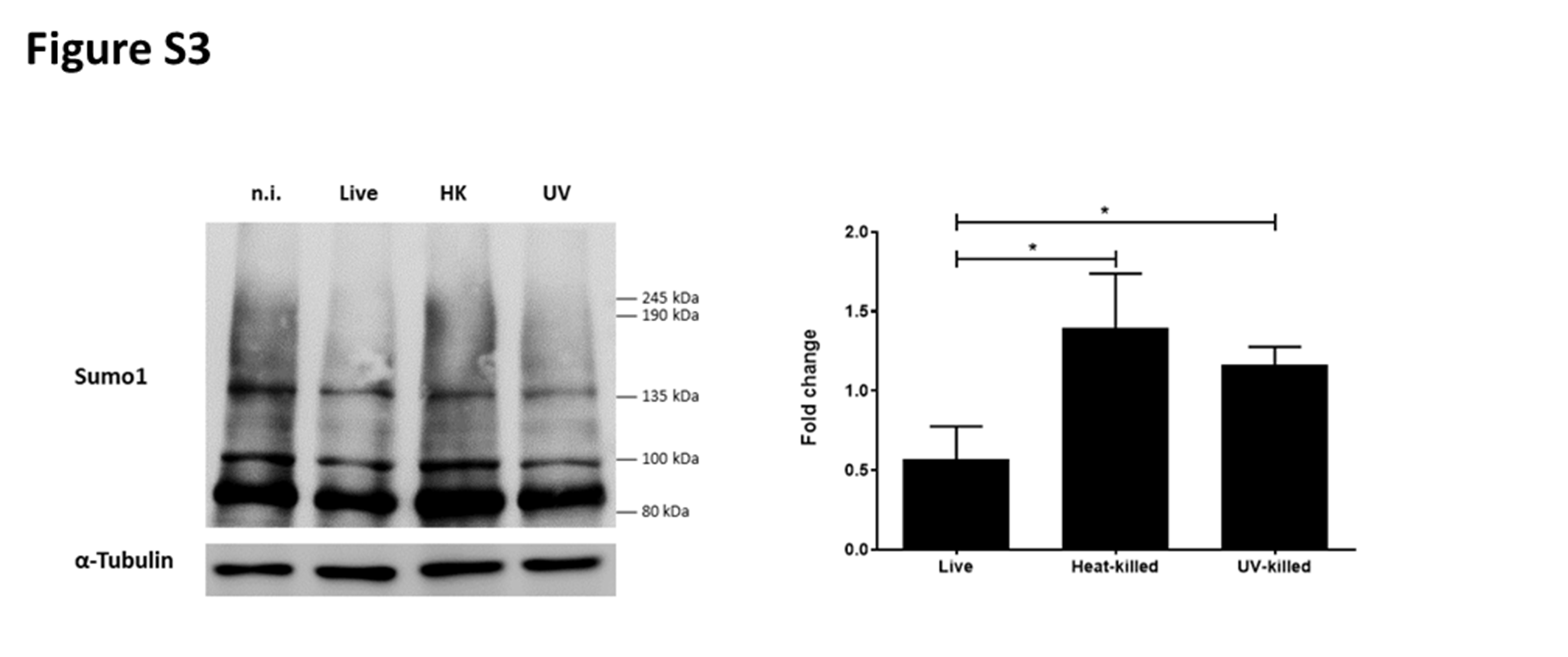

Supplement: FIG S3 [file mBio.01733-20-sf003.tif]

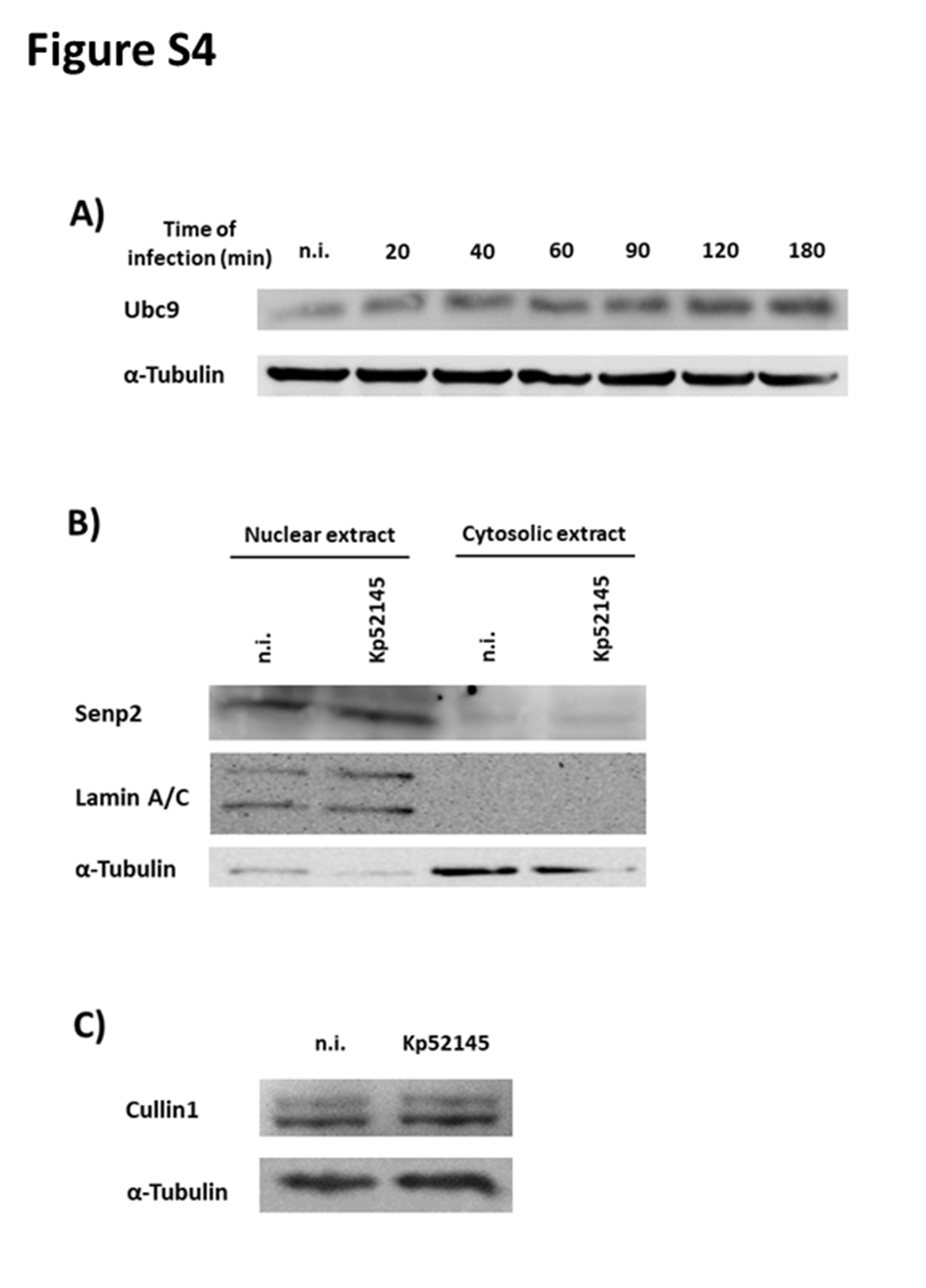

Supplement: FIG S4 [file mBio.01733-20-sf004.tif]

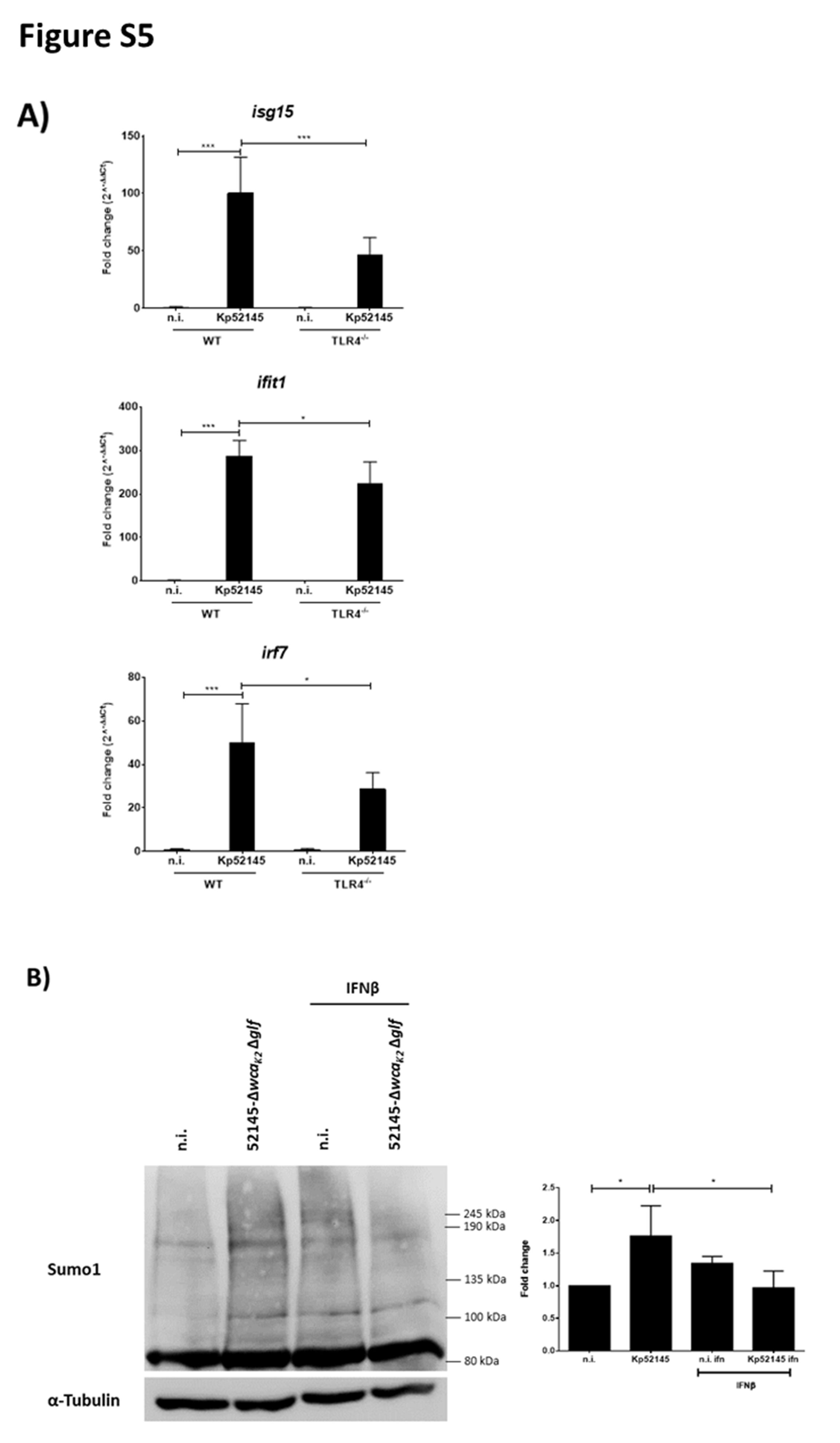

Supplement: FIG S5 [file mBio.01733-20-sf005.tif]

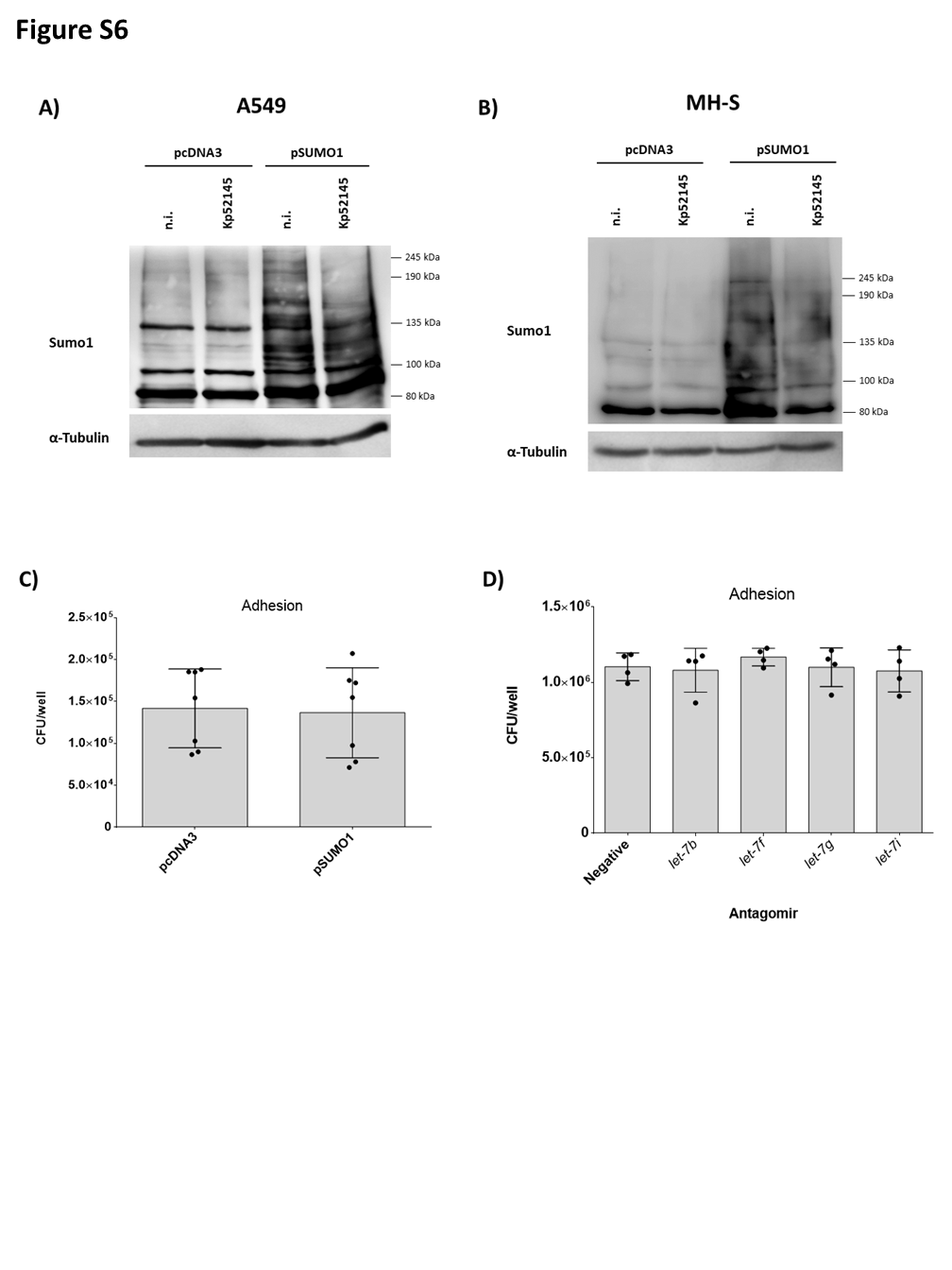

Supplement: FIG S6 [file mBio.01733-20-sf006.tif]

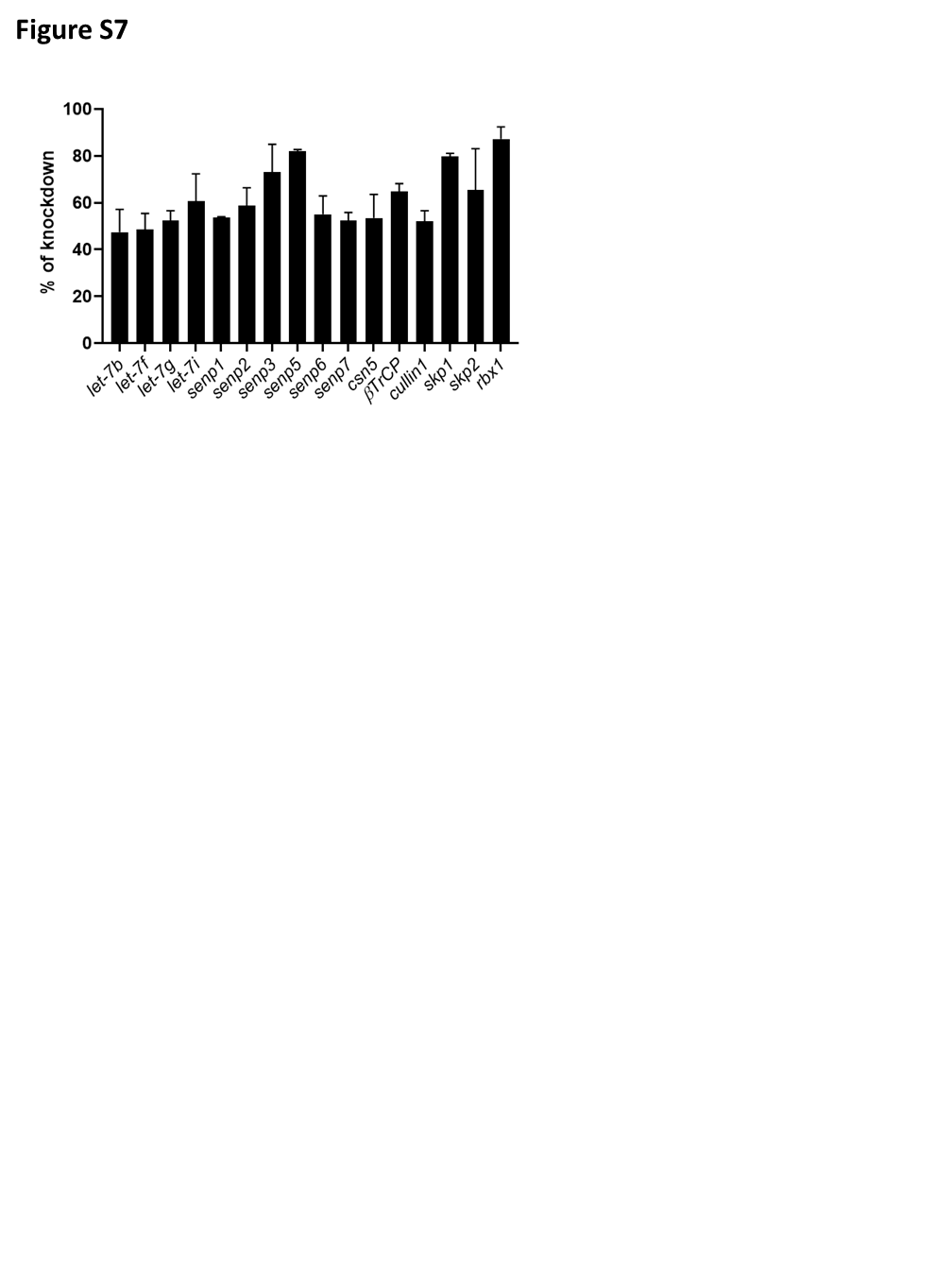

Supplement: FIG S7 [file mBio.01733-20-sf007.tif]
