# Supplementary material for: Klebsiella pneumoniae Reduces SUMOylation To Limit Host Defense Responses
Source: mBio. 2020 Sep 29;11(5):e01733-20. doi: 10.1128/mBio.01733-20 (PMC7527722; doi:10.1128/mBio.01733-20)
Supplement: TABLE S1 [file mBio.01733-20-st001.docx]

**Supplementary Table 1.** List of primers used in this work

| **Name** | **Sequence (5’-3’)** |
| --- | --- |
| *mlet-7a-5p* forward | gcagtgaggtagtaggttg |
| *mlet-7a-5p* reverse | ggtccagtttttttttttttttaactatac |
| *mlet-7b-5p* forward | cagtgaggtagtaggttgtgt |
| *mlet-7b-5p* reverse | ggtccagtttttttttttttttaacca |
| *mlet-7c-5p* forward | gcagtgaggtagtaggttgt |
| *mlet-7c-5p* reverse | ggtccagtttttttttttttttaacca |
| *mlet-7d-5p* forward | cgcagagaggtagtaggttg |
| *mlet-7d-5p* reverse | ggtccagtttttttttttttttaactatg |
| *mlet-7e-5p* forward | gcagtgaggtaggaggttg |
| *mlet-7e-5p* reverse | ggtccagtttttttttttttttaactatac |
| *mlet-7f-5p* forward | cgcagtgaggtagtagattg |
| *mlet-7f-5p* reverse | caggtccagtttttttttttttttaac |
| *mlet-7g-5p* forward | cgcagtgaggtagtagtttg |
| *mlet-7g-5p* reverse | caggtccagtttttttttttttttaac |
| *mlet-7i-5p* forward | gcagtgaggtagtagtttgtg |
| *mlet-7i-5p* reverse | ggtccagtttttttttttttttaacag |
| *snoRNA-202* forward | AGTACTTTTGAACCCTTTTCCA |
| *snoRNA-202* reverse | GTCGTATCCAGTGCAGGGTCCGAGGTATTCGCACTGGATACGACCATCAG |
| m*HPRT* forward | GATCAGTCAACGGGGGACAT |
| m*HPRT* reverse | GGTCCTTTTCACCAGCAAGC |
| m*ifnβ* forward | ATGGTGGTCCGAGCAGAGAT |
| m*ifnβ* reverse | CCACCACTCATTCTGAGG |
| m*ifit1* forward | CAGGTTTCTGAGGAGTTCTG |
| m*ifit1* reverse | TGAAGCAGATTCTCCATGAC |
| m*isg15* forward | GGGGCCACAGCAACATCTAT |
| m*isg15* reverse | CGCTGGGACACCTTCTTCTT |
| m*irf7* forward | TTGGATCTACTGTGGGCCCA |
| m*irf7* reverse | CTTGCCAGAAATGATCCTGGG |
| h*GAPDH* forward | GAGAAGGCTGGGGCTCATTT |
| h*GAPDH* reverse | AGTGATGGCATGGACTGTGG |
| h*CSN5* forward | AAGAAATCCTGGCGGCGAA |
| h*CSN5* reverse | GCATCAGACCCATCACTTCCA |
| h*senp1* forward | TCTGTTCTTTGACCTCCTGCCC |
| h*senp1* reverse | AGTCACTTCTCCAGCATCCATC |
| h*senp2* forward | CTTGTGAACTGACAGGTTCTGG |
| h*senp2* reverse | ACCAAAGGAAGGCAGGACTC |
| h*senp3* forward | ACGTGCTGACCATGGATGAC |
| h*senp3* reverse | GGTCCACCTTTTCACCCCAT |
| h*senp5* forward | TTCACCTGGAAGTCCACTGGT |
| h*senp5* reverse | CAGTCTGCCAACCCTGAAGA |
| h*senp6* forward | GCAGCCCAAAGGAAAGAATACC |
| h*senp6* reverse | AGGCTCCACTTGTGATTCGG |
| h*senp7* forward | TCCGAGGGTGTCCTGTTACT |
| h*senp7* reverse | TGTCGAAGGCAATGAGTCTGA |
